# Foundations for an ontology of brain areas, circuits and functions

**DOI:** 10.1186/1471-2202-14-S1-P19

**Published:** 2013-07-08

**Authors:** Bénédicte M Batrancourt

**Affiliations:** 1 CRICM UPMC/INSERM UMR_S975, Paris, 75013, France

## 

This article describes a method for building the foundations for an ontology of brain areas, brain circuits and cerebral functions. This ontology challenges the complex pattern of neural connections that underlie cognition and behavior. The purpose of this ontology is to explore the structural foundations of human brain function. The ontology is composed from three main modules. The first one concerns the brain anatomy and more exactly the brain areas (e.g. 'the frontal lobe', 'the inferior frontal gyrus'), the second one concerns the brain circuits (e.g. 'the arcuate fasciculus connecting Wernicke's and Broca's areas') and finally the last one which concerns the cognitive anatomy (e.g. 'the conduction aphasia is interpreted as a disconnection between the temporal and frontal brain language areas').

We built **the brain anatomy module (i.e., the brain areas module) **using the BrainInfo resource [1] and more exactly NeuroNames, an ontology designed to compensate for ambiguities in neuroanatomical nomenclature. NeuroNames recognizes a very large set of names of neuroanatomical entities and accommodates synonyms and homonyms of the standard terms in many languages. It defines complex structures as models composed of primary structures, which are defined in unambiguous operational terms [2]. NeuroNames is a resource vocabulary of the NLM's Unified Medical Language System (UMLS, 2011) and the basis for the brain regions component of NIFSTD [3]. The brain anatomy module contains 2900 concepts extracted from Neuronames including the primary cerebral structures, superstructures and related superficial features and others ways subdividing the brain. We did not hold for this study, the hierarchical relations of each structure to its superstructures and substructures providing by Neuronames because the links will be established as a connected and more topological way.

We are currently building **the brain circuits module **using the Atlas of Human Brain Connections [3] which capitalizes on diffusion MRI tractography methods to provide a comprehensive overview of connections derived from virtual in vivo tractography dissections of the human brain. This atlas details descriptions of the major white matter connections, their function, and associated clinical syndromes.

We will build the **function module **as a summary and a anatomo-functional synthesis amongst the previous modules. We will retain two entries: the normal functioning (e.g., the language function) and the pathological entry entry (e.g., the aphasia). This issue will consist to associate a function (and dysfunction) to a specific circuit and linked areas using authoritative neuroscience textbooks (e.g. Geschwind attributed the arcuate fasciculus the main role in speech repetition disturbances and resulting in the so-called Wernicke-Geschwind model of language).

We maintain two manifestations of the ontology. The first manifestation is specified in the formal Web Ontology Language OWL. The second one is specified in a graph format. To visualize the graphs we use Gephi [4], which is an interactive visualization and exploration platform for all kind of networks and complex systems, dynamic and hierarchical graphs.
